# Correlation of Gli1 and HER2 expression in gastric cancer: Identification of novel target

**DOI:** 10.1038/s41598-017-17435-6

**Published:** 2018-01-10

**Authors:** Xinyu Shao, Xiaoyi Kuai, Zhi Pang, Liping Zhang, Longyun Wu, Lijuan Xu, Chunli Zhou

**Affiliations:** The Affiliated Suzhou Hospital of Nanjing Medical University, Suzhou, 215006 P.R. China

## Abstract

HER2 becomes the standard of care for guiding adjuvant treatment of gastric cancer with trastuzumab in recent years. However, the usage of this target agent is still limited because of the resistance to trastuzumab or the negative expression of HER2 in tumor tissues. The Gli1 and HER2 both play an important role in the pathogenesis of gastric cancer. However, the correlation of them is still unclear. Here we found Gli1 and HER2 are highly expressed in gastric cancer tissues, and they are positively related. Next, we found Gli1 positive patients live a shorter survival time no matter HER2 positive or negative. Furthermore, univariate and multivariate analysis revealed that venous invasion, HER2 expression, Gli1 expression were independent prognostic factors for the survival time in gastric cancer. In addition, suppressing the expression level of Gli1 can decrease the cell viability and migration ability in cells and subcutaneous tumors. Finally, we found that HER2 may regulate Gli1 by Akt–mTOR–p70S6K pathway. Inhibit of HER2 and SMO have synergistic effect on reduction of cell viability. In conclusion, Gli1 is a favorable prognostic indicator in gastric cancer. As a novel target, Gli1 worth further study, especially in Her2-targeted therapy-resistant cancers.

## Introduction

Cancer is the leading cause of death among men aged 45 to 79 in recent 5 years in US^1^. In 2012, there were 951,000 cases of gastric cancer worldwide, the highest regional rates were in Eastern/Southeastern Asia^2^. *H*. *pylori* infection^3^, environmental problems and inherited genes all contribute to gastric oncogenesis. Nowadays, the complex therapies including surgery, chemotherapy and molecular targeted therapy have been used widely. However, advanced gastric cancers still face poor therapeutic effect and terrible drug resistance^4–6^. Therefore, it is very urgent to identify novel therapeutic targets to treat this fatal disease.

The Hedgehog (HH) pathway plays a crucial role in cell proliferation and metastasis in human tumors^7–10^. It has three legends, Sonic Hedgehog (SHH), Indian Hedgehog (IHH) and Desert Hedgehog (DHH) in classical HH signaling pathway. When the pathway is activated, the HHs bind to 12-transmembrane receptor Patched (PTCH) and then depresses it. The inhibition of PTCH resulted in the release the restraining of Smoothened (SMO, a G-coupled receptor-like protein). Then SMO translocates to the primary cilium and phosphorylate SUFU. Subsequently, Gli zinc-finger transcription factors, including Gli1, Gli2 and Gli3, translocate into nucleus and induce the regulatory expression of HH target genes^11–13^. Emerging researches have suggested that Hedgehog pathway is closely associated with poor prognosis and drug resistance of cancer^14–16^.

Overexpression of human epidermal growth factor receptor 2 (HER2) in gastric cancer is related to poor outcome^17,18^. Because the HER2 positive gastric cancer patients receive significant benefit from trastuzumab, testing the expression of HER2 before targeted therapy is necessary^19^. With the more and more severe condition of drug resistance of trastuzumab, the way to prolong survival time of gastric cancer patients is limited. New studies have suggested that the expression of Gli1 is related to prognosis in HER2 positive breast cancer, and Gli1 is regulated by HER2 via PI3K-Akt pathway in esophageal adenocarcinoma^20,21^. That is to say, inhibiting Hedgehog pathway, especially targets Gli1, may become one of the effective treatments of this lethal disease. However, only few investigations were done to explore the relationship of Gli1 and HER2 in gastric cancer. And there are fewer studies targeting Gli1 according to their relationship. In this study, we tested the expression level of Gli1 and HER2 in specimens, and analyzed their relationship through cell researches. We retrospectively evaluated the impact of Gli1 on the outcomes of gastric cancer patients. Furthermore, we validated the anti-tumor effects of down-regulation of Gli1 in gastric cancer cells and in heterologous subcutaneous caner mice, trying to identify a novel target for therapeutic intervention, especially in HER2-targeted therapy-resistant gastric cancer.

## Results

### Expression levels of Gli1/HER2 in human gastric cancer tissues and paired adjacent tissues

**Figure 1:**
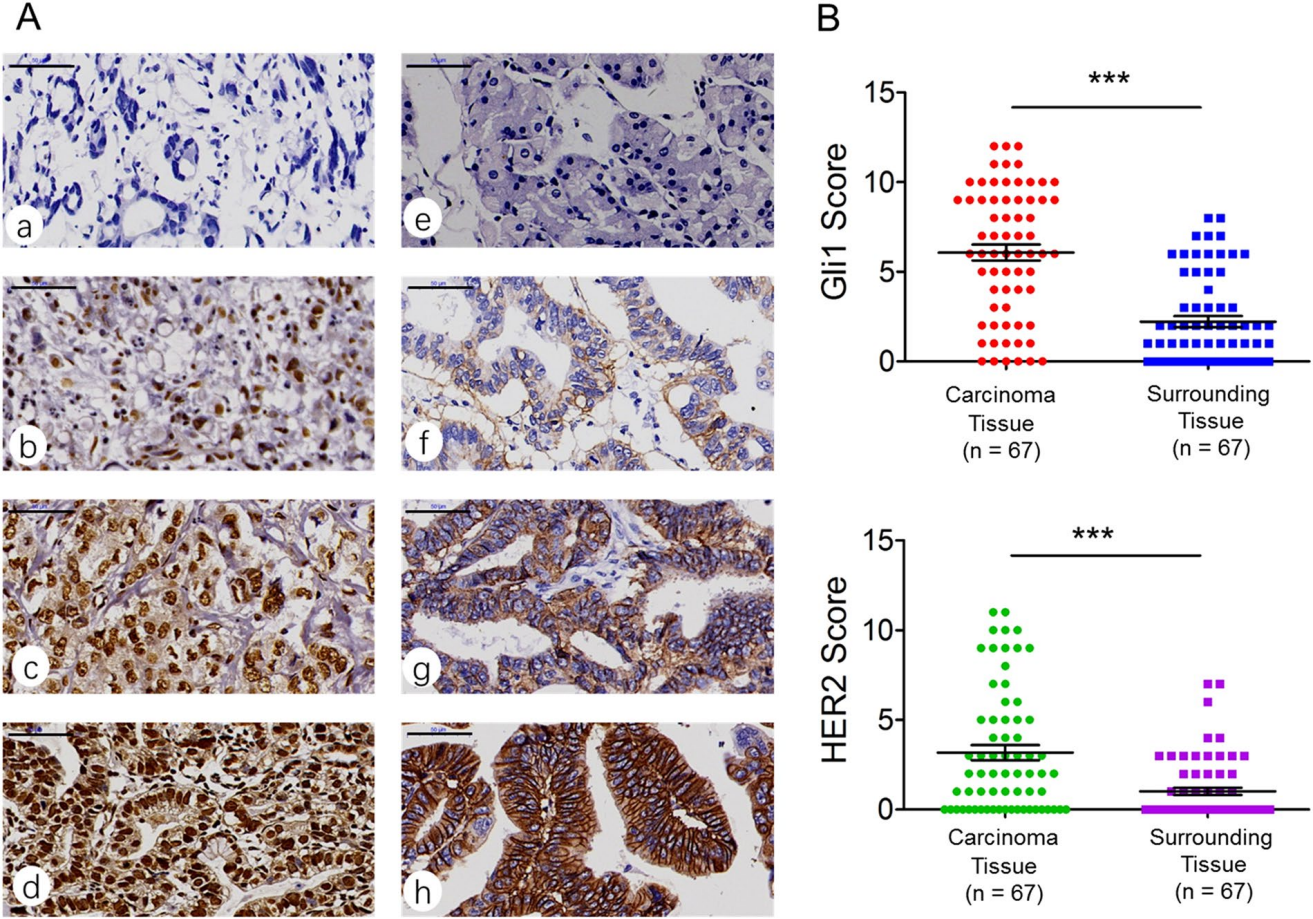
Expression of Gli1 and HER2 in gastric cancer tissues. (**A**) Immunohistochemical staining of Gli1 and HER2 in 67 human gastric cancer tissues and surrounding tissues(200×). The protein expression of Gli1 was negative (a), weak (b), positive (c), strong positive (d), and the protein expression of HER2 was negative (e), weak (f), positive (g), strong positive (h), respectively. (**B**) Staining scores analysis of Gli1 and HER2 expression in 67 cancer tissue samples and their surrounding tissues. ***P < 0.001.

We detected the expression level of Gli1 and HER2 in the 67 primary human gastric cancer tissues and paired adjacent healthy tissues by immunohistochemistry (IHC). The percentage and intensity scores were then multiplied to obtain a total staining score (Fig. 1A,B). We judged positive or negative according to the rule mentioned above. After IHC staining, staining scores of Gli1 and HER2 were calculated. The result showed the expression levels of Gli1 and HER2 were significantly higher in gastric cancer than in para-cancer tissues *(P* < 0.001 and *P* < 0.001, Fig. 1B). Thus, we concluded that Gli1 and HER2 were overexpressed in gastric cancer tissues. For further study, we investigated whether Gli1 correlates with HER2 expression in tissues. By IHC, we found Gli1 was positively expressed in 17 out of 20 HER2 positive tumor tissues, while in 28 out of 47 HER2 negative tumors tissues, fourfold table chi-square test showed the expression level of Gli1 and HER2 was related (*P* = 0.043, Table 1).

**Table 1:** Statistics of HER2 and Gli1 expression in human gastric cancer tissues.

	HER2 positive	HER2 negative	*P* value
Gli1 positive	17	28	0.043*
Gli1 negative	3	19	

*P < 0.05.

### Association between Gli1/HER2 expression levels and the clinicopathological factors

We accessed whether the expression level of Gli1 or HER2 is associated with clinicopathological factors of postoperative gastric cancer patients (Table 2). Pearson χ2 test analysis showed that expression level of Gli1 is correlated to the depth of tumor invasion (*P* < 0.001), lymph node metastasis (*P* = 0.021) and TNM staging (*P* = 0.003). And, HER2 had a significant relationship with depth of tumor invasion (*P* = 0.013), lymph node metastasis (*P* = 0.014) and TNM staging (*P* = 0.006). There is no correlation between Gli1/HER2 and other clinicpathological parameters such as age, gender or size of tumor (*P* > 0.05).

**Table 2:** Association between Gli1/HER2 and clinic-pathological factors in 67 patients with gastric cancer.

	Gli1			HER2		
	Negative	Positive	*P*	Negative	Positive	*P*
Age(years)						
≤60	9	16	0.670	17	8	0.767
>60	13	29		30	12	
Gender						
Male	18	32	0.344	35	15	0.963
Female	4	13		12	5	
Size(cm)						
≤5	13	27	0.943	26	14	0.262
>5	9	18		21	6	
Depth of tumor invasion						
T1-2	10	2	<0.001***	12	0	0.013*
T3-4	12	43		35	20	
Degree of differentiation						
Well	10	22	0.792	24	8	0.407
Poor	12	23		23	12	
Lymph node metastasis						
Yes	11	35	0.021*	28	18	0.014*
No	11	10		19	2	
TNM staging						
I-II	13	10	0.003**	21	2	0.006**
III-IV	9	35		26	18	

*P < 0.05, **P < 0.01, ***P < 0.001.

### Gli1 expression links with gastrectomy patients’ survival

**Figure 2:**
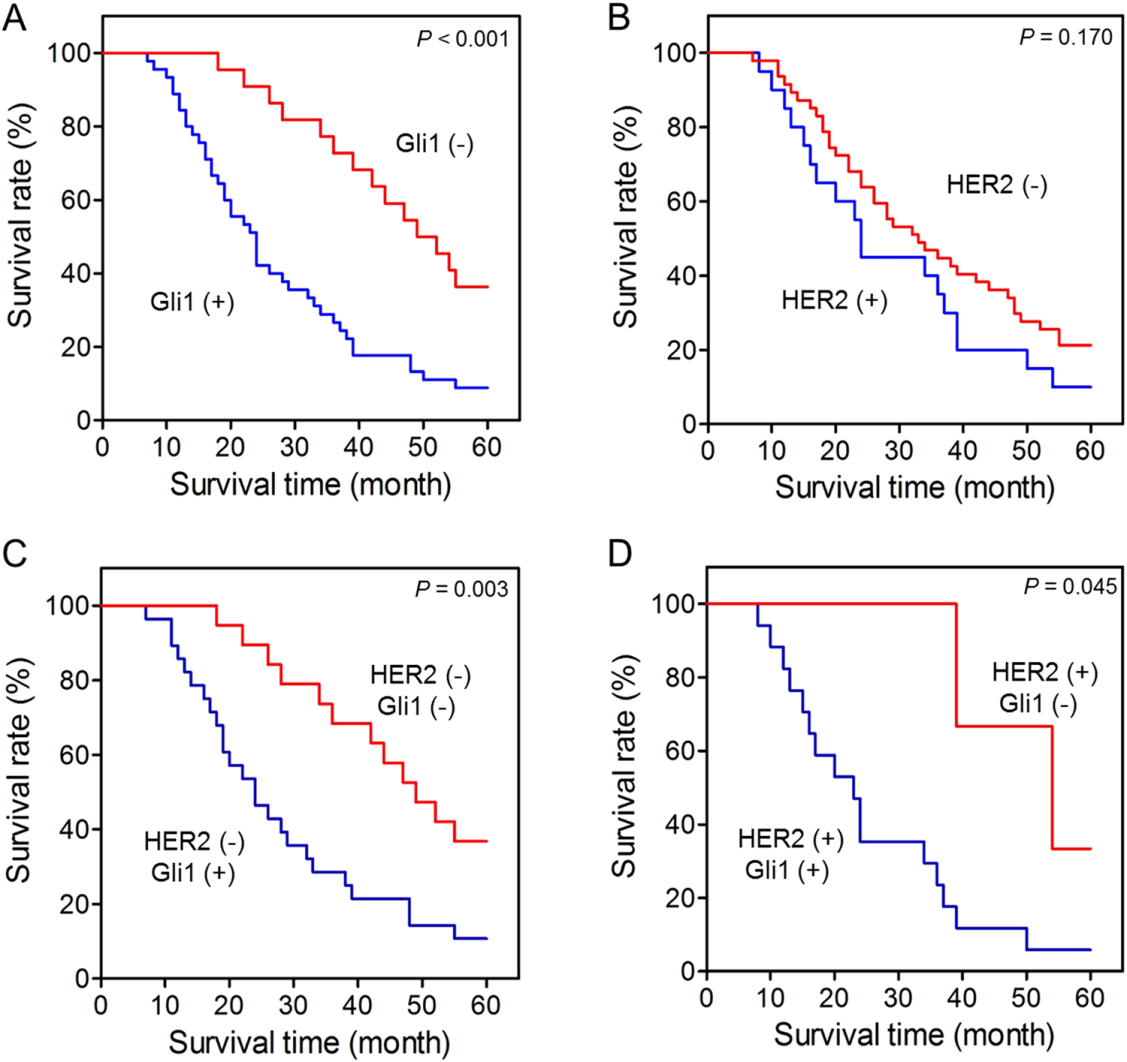
Kaplan-Meier survival analysis in patients after gastrectomy. (**A**) 67 gastric cancer patients after operation were analyzed according to the Gli1 expression. In this 67 patients, Gli1 positive expression was 45 and negative expression was 22. (**B**) 67 gastric cancer patients after operation were analyzed according to the HER2 expression. HER2 positive expression was 20 and negative expression was 47. (**C**) 47 gastric cancer patients with negative HER2 expression after operation were analyzed according to the Gli1 expression. Gli1 positive expression was 28 and negative expression was 19 in patients. (**D**) 20 gastric cancer patients with positive HER2 expression after operation were analyzed according to the Gli1 expression. Gli1 positive expression was 17 and negative expression was 3 in patients.

To elaborate whether the expression levels of Gli1/HER2 in gastric cancer tissues exert an influence on overall survival of patients after gastrectomy, we analyzed survival curves according to target genes expression. In result, the survival of patients with positive Gli1 expression was worse than those with negative Gli1 expression (*P* < 0.001, Fig. 2A). Whereas the patients with negative HER2 got a modest survival benefit, which didn’t reach a significant difference (*P* = 0.170, Fig. 2B). Based on the expression level of HER2, we further analyzed the association between Gli1 expression and survival of patients. As the outcome shows, no matter the expression of HER2 was positive or negative, the survival of gastric cancer patients with positive Gli1 expression is poorer than the patients with negative ones (*P* = 0.003, *P* = 0.045, Fig. 2C,D). Univariate analysis revealed that Gli1 expression, venous invasion, TNM staging, depth of tumor invasion, lymph node metastasis, size of tumor, differentiation, neural invasion were associated with an inferior survival duration (*P* < 0.05, Table 3). However, only venous invasion, HER2 expression, Gli1 expression were verified to be independent prognostic factors for the survival in gastric cancer patients after multivariate analysis (*P* < 0.05, Table 3), especially Gli1 expression (*P* < 0.001, Table 3).

**Table 3:** Results of univariate and multivariate analyses of gastrectomy patients’ survival by Cox’s proportional hazard model.

Factor	Group	Number of patients	Univariate analysis		Multivariate analysis	
			HR (95% CI)	*P* value (log-rank)	HR (95% CI)	*P* value (log-rank)
Age	≤60 years	25	1		1	
	>60 years	42	1.196 (0.693–2.064)	0.520	1.360 (0.681–2.715)	0.384
Gender	Male	50	1		1	
	Female	17	1.320 (0.719–2.424)	0.370	2.118 (0.894–5.019)	0.088
Size of tumor	≤5 cm	38	1		1	
	>5 cm	29	0.428 (0.246–0.745)	0.003**	1.089 (0.563–2.108)	0.800
Depth of tumor invasion	T1-2	12	1		1	
	T3-4	55	0.183 (0.072–0.465)	<0.001***	1.138 (0.257–5.038)	0.865
Lymph node metastasis	Negative	21	1		1	
	Positive	46	0.123 (0.058–0.258)	<0.001***	0.222 (0.026–1.882)	0.168
Differentiation	Well	36	1		1	
	Poor	31	2.255 (1.288–3.947)	0.004**	0.746 (0.315–1.766)	0.505
Venous invasion	Negative	38	1		1	
	Positive	29	0.361 (0.208–0.627)	<0.001***	0.357 (0.171–0.745)	0.006**
Neural invasion	Negative	42	1		1	
	Positive	25	0.453 (0.262–0.781)	0.004**	0.516 (0.255–1.047)	0.067
TNM staging	I - II	23	1		1	
	III - IV	44	0.121 (0.060–0.245)	<0.001***	0.233 (0.022–2.423)	0.223
HER2 expression	Negative	47	1		1	
	Positive	20	0.678 (0.385–1.194)	0.178	2.200 (1.090–4.442)	0.028*
GLI1 expression	Negative	22	1		1	
	Positive	45	0.332 (0.179–0.615)	<0.001***	0.210 (0.093–0.473)	<0.001***

**Figure 3:**
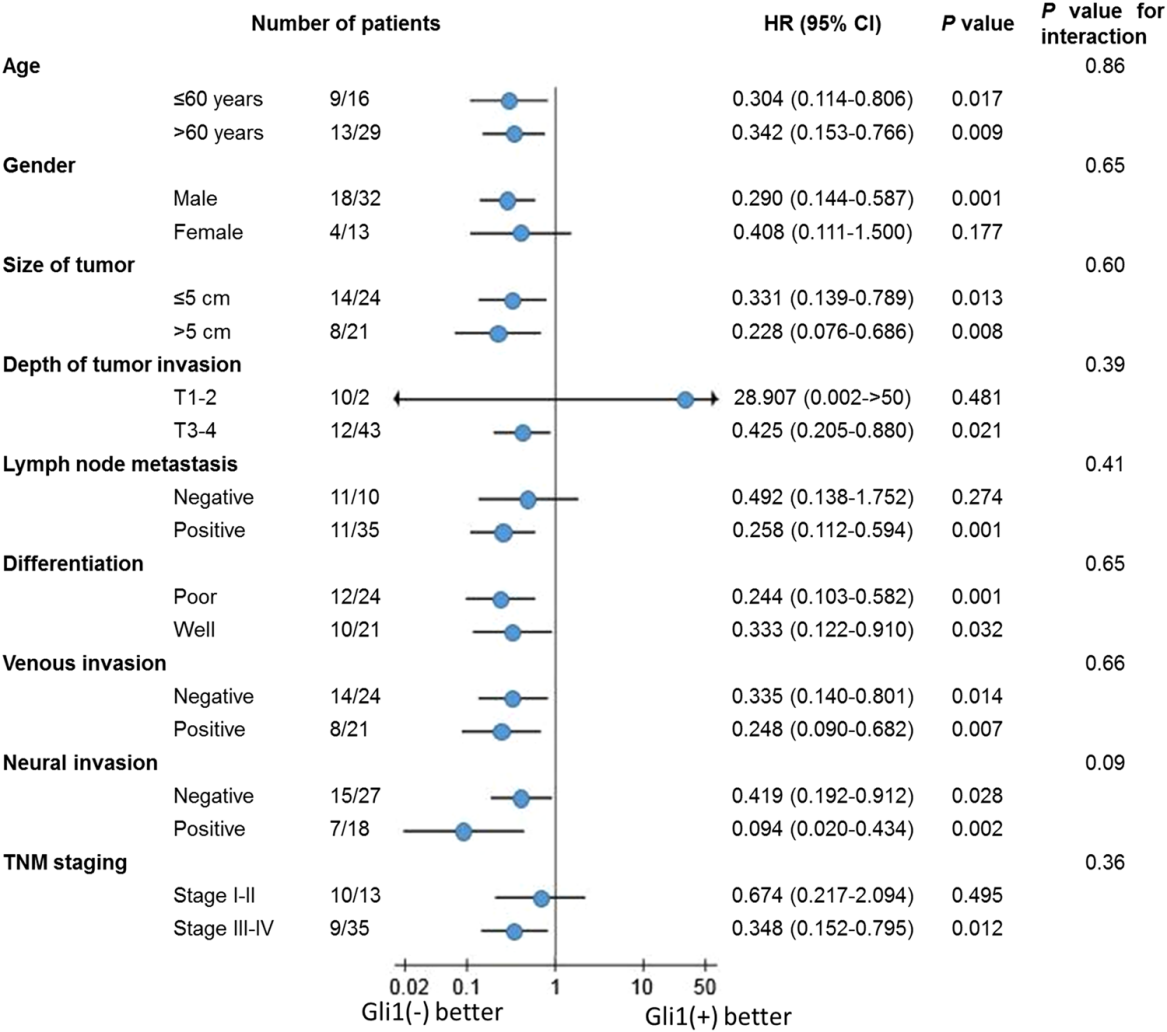
Subgroup analysis for the influence factor of survival duration of gastrectomy patients according to Gli1 expression. There were 9 Gli1 negative patients and 16 Gli1 positive patients in the gastric cancer patients after operation who aged under 60 years old. The expression level of Gli1 was associated with the survival duration in patients under 60 years old (*P* = 0.017), so as in patients over 60 years old (*P* = 0.009). The same in other scale such as gender, the size of tumor, depth of tumor invasion, lymph node metastasis, differentiation, venous invasion, neural invasion and TNM staging.

To further explore the influence of different Gli1 expression on other prognostic factors, we performed a subgroup analysis. The results showed that Gli1 expression significantly shortened the survival no matter the patients’ age, the size of tumor, differentiation, venous invasion or neural invasion (*P* > 0.05, Fig. 3). For male patients, the survival time was worse in those with positive Gli1 expression than female patients. Meanwhile, Gli1 expression affected the survival in patients with stage T3-T4 (*P* = 0.021, Fig. 3), positive lymph node metastasis (*P* = 0.001, Fig. 3) and stage III-IV (*P* = 0.012, Fig. 3), indicating that patients at the same depth of tumor invasion (stage T3-T4), lymph node metastasis (positive) or same TNM stage (stage III-IV), patients with Gli1 positive expression could have a significant poorer survival than others.

### Gli1 depleted depresses the growth of gastric cancer *in vivo* and *in vitro*

**Figure 4:**
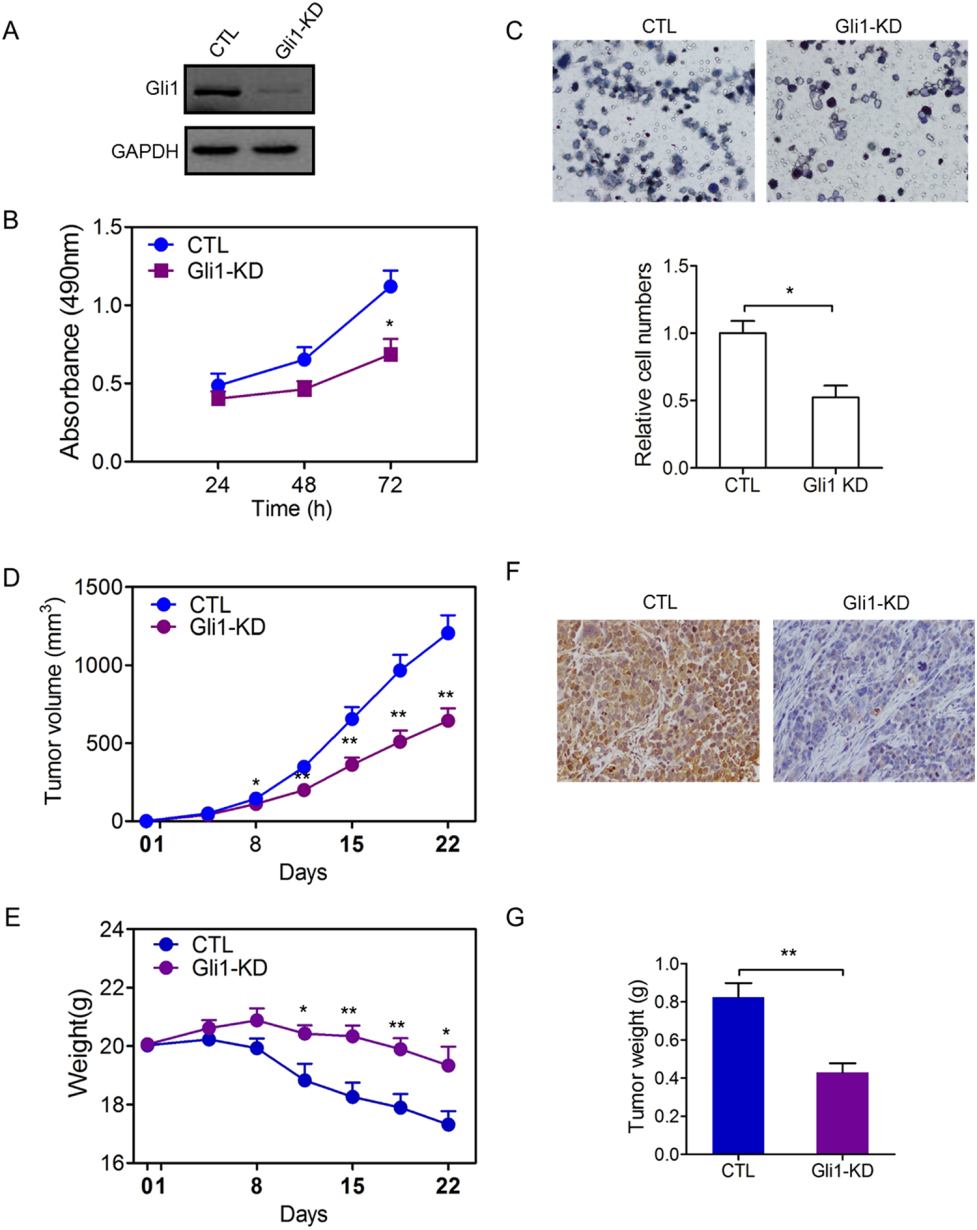
Knockdown of Gli1 suppressing gastric tumor growth *in vitro* and *in vivo*. (**A**) SGC7901 cells were transfected with negative control shRNA (CTL) or Gli1 shRNA (Gli1-KD). The protein was analyzed by Western blot. (**B**) MTT assay was used to test the proliferation ability of cells transfected with negative control shRNA (CTL) or Gli1 shRNA (Gli1-KD), presented as mean ± S.E.M. (n = 3). (**C**) Transwell assay was used to test the migration ability of cells transfected negative control shRNA (CTL) or Gli1 shRNA (Gli1-KD). Representative photographs were presented (X100) and the relative number of migratory cells were counted as mean ± S.E.M. (n = 5). (D-G) SGC7901 cells transfected with negative control shRNA (CTL) or Gli1 shRNA (Gli1-KD) stably were transplanted into nude mice (n = 8). (**D**) The volume of the tumors was measured twice a week during the indicated 3 weeks. The tumor mass of each group was presented as mean ± S.E.M. (n = 8). (**E**) The weight of mice was measured twice a week during the indicated 3 weeks. The weight of mice in each group was presented as mean ± S.E.M. (n = 8). (**F**) Tumor was tested by immunohistochemical staining of Gli1 in each group after mice killed. (**G**) The weight of tumor mass in each group was also presented. Data was presented as mean ± S.E.M. (n = 8). *P < 0.05, **P < 0.01, ***P < 0.001.

As described above, the expression level of Gli1 is an independent prognostic factor of the survival duration of gastric cancer patients after operation. As a new target, Gli1 shows a cheerful prospect. To confirm the finding that decreasing the expression of Gli1 can further inhibit the tumorgenesis, we did studies both *in vivo* and *in vitro*. The efficiency of Gli1 knockdown in the two groups was shown in Fig. 4A. We transfected SGC7901 cells with control-shRNA or Gli1-shRNA. Compared with control group, the cells showed decreased cell viability and migration ability after knockdown Gli1 (Fig. 4B,C). Furthermore, we evaluated the effect of Gli1 in a mice model. We selected 3-4 week nude mice. We implanted the same number of SGC7901 gastric cancer cells, which treated differently with control-shRNA or Gli1-shRNA onto the subcutaneous sites of mice and measured tumor size twice a week. Then, we found that the subcutaneous tumors treated with Gli1 depletion grow much slower and relatively get a more steady weight (Fig. 4D–F). After stripping the subcutaneous tumors, we accessed the weight of tumors and finding the tumors derived from Gli1 depleted group were decreased markedly (*P* < 0.01, Fig. 4G).

### Gli1 Crosstalk with HER2 through AKT-mTOR pathway

**Figure 5:**
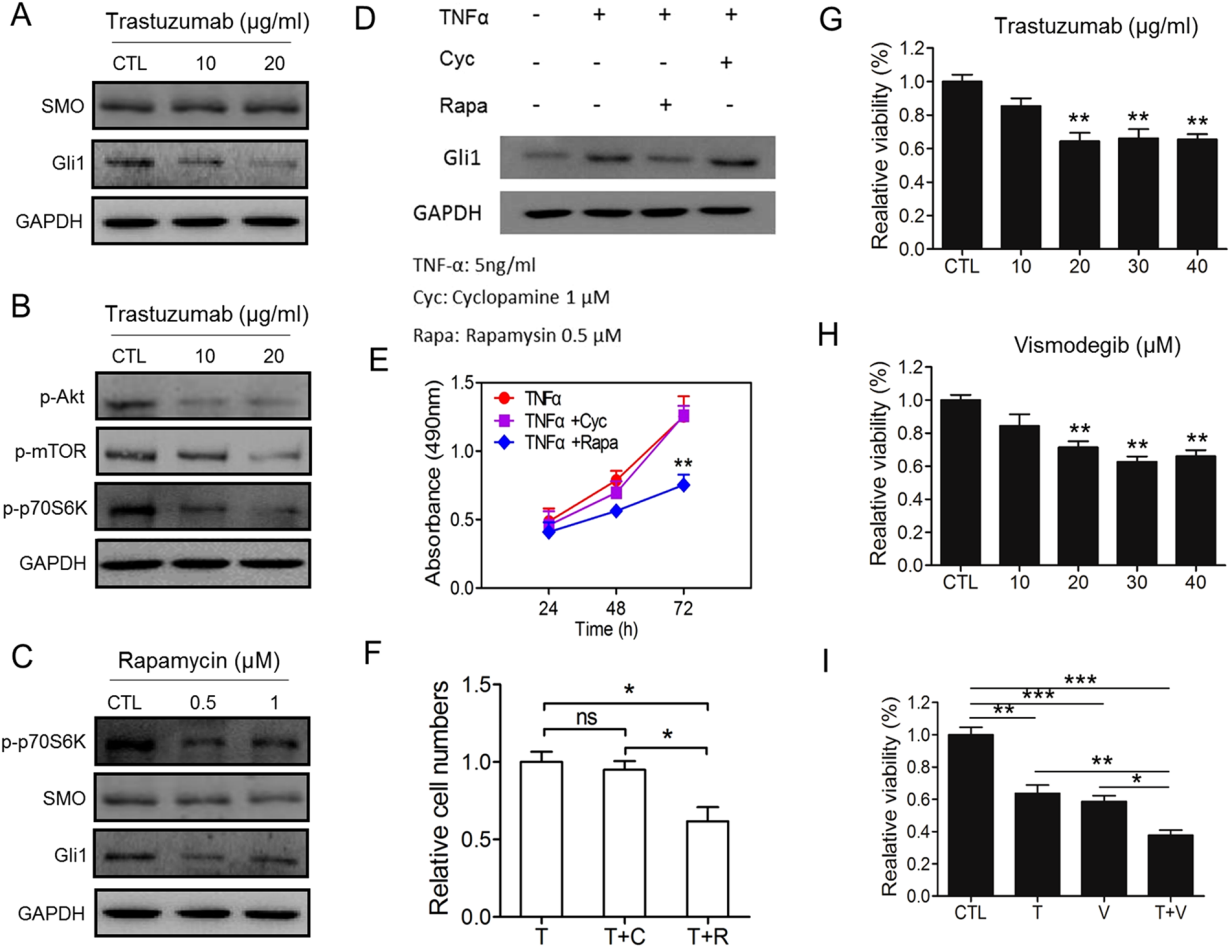
HER2 regulates Gli1 via Akt-mTOR pathway, combination treatment of trastuzumab and vismodegib obtained a synergistic effect. (**A**) The expression of SMO and Gli1 was investigated via Western blotting after SGC7901 treated with Trastuzumab (the HER2 inhibitor) for 48 h. (**B**) The expression of p-Akt, p-mTOR, p-70S6K was tested by Western blotting after SGC7901 treated with Trastuzumab for 48 h. (**C**) The expression of p-70S6K, SMO and Gli1 was detected via Western blotting after SGC7901 treated with rapamycin (The mTORC1 inhibitor) for 48 h. (**D**) The expression of Gli1 was detected via Western blotting after SGC7901 were treated with TNFα (5ng/ml), TNFα (5ng/ml) and cyclopamine (1 μM) or TNFα (5 ng/ml) and rapamycin (0.5 μM) for 24 h. (**E**) MTT assay was used to test the proliferation ability of cells treated wih TNFα (5ng/ml), TNFα (5ng/ml) and cyclopamine (1 μM) or TNFα (5 ng/ml) and rapamycin (0.5 μM) for 24 h, 48 h and 72 h,presented as mean ± S.E.M. (n = 3) (**P < 0.01). (**F**) Transwell assay was used to test the migration ability of cells treated with TNFα (5ng/ml) (T), TNFα (5ng/ml) and cyclopamine (1 μM) (T + C) or TNFα (5 ng/ml) and rapamycin (0.5 μM) (T + R) for 24 h. (**G**) Trastuzumab reduced the viability of SGC7901 significantly. The cells treatED with different concentrations of trastuzumab for 48 h. (**H**) Vismodegib(the SMO inhibitor) reduced the viability of SGC7901 significantly. The cells treatment with different concentrations of vismodegib for 48 h. (**I**) The combined or single treatment of trastuzumab and vismodegib for SGC7901 was analyzed. *P < 0.05, **P < 0.01, ***P < 0.001.

We found there is a significant association between Gli1 and HER2 expression in gastric cancer specimens. Thus, we further analyzed their relationship in cell lines. As a first step, we select a gastric cancer cell line, SGC7901 in our study. We treated SGC7901 with trastuzumab (the HER2 inhibitor) for 48 hours at the concentration of 10 or 20 μg/ml respectively. Compared with the control group, except SMO (Gli1 upstream target), the expression level of Gli1, p-AKT, p-mTOR and p-p70S6k (mTOR downstream target) decreased obviously (Fig. 5A,B). The quantitative statistics see Supplementary Fig. S1A,B. In order to further explore the result, we detected the association between Gli1 and Akt-mTOR, the downstream pathway of HER2. Some studies revealed there is a crosstalk between mTOR and Gli1^22^. We used rapamycin, the specific mTORC1 inhibitor, to treated SGC7901 for 48 hours and found the protein expression of Gli1 decreased distinctly, but SMO, the upstream of Gli1, was unchanged(Fig. 5C). The quantitative statistics see Supplementary Fig. S1C. To further confirm the result, SGC7901 was treated with TNFα, which reported could enhance the expression of Gli1 via mTOR–p-70S6K-Gli1 axis^22^. Western blot, proliferation and migration ability assays showed rapamysim could reverse the function of TNFα, while cyclopamine (a canonical hedgehog pathway inhibitor target on SMO) could not (Fig. 5D–F). The transwell picture see Supplementary Fig. S1D. These researches suggested that HER2 may regulate Gli1 by Akt–mTOR–p-70S6K pathway, not canonical SMO-Gli1 pathway.

### Inhibit of HER2 and SMO has synergistic effect on reduction of cell viability

Next step, we tested the impact of trastuzumab and Vismodegib (the SMO inhibitor) on the viability of SGC7901. After a 48 treatment, cells treated with trastuzumab reached a reduction plateau of cell viability at the concentration of 20 μg/ml (Fig. 5G). And the reduction plateau of Vismodegib was 20 μmol/l (Fig. 5H). Whereas, the reduction plateau of the dose response curve decreased obviously when we combined trastuzumab and Vismodegib to treat the cells (Fig. 5I). That is to say, inhibit Gli1 both via HER2–Akt–mTOR–p-70S6K and Hedgehog pathway has synergistic effect on gastric cancer cells.

## Discussion

Gastric cancer still faces poor prognosis and low survival rate in the world, especially in Asia^23^. Although the improvement of surgical techniques and the widely use of chemotherapies prolong the survival rate modestly, gastric cancer patients still face diverse side effects and severe drug-resistance^24^. Testing the expression of HER2 becomes the standard of care for guiding adjuvant treatment of gastric cancer with trastuzumab^19,25,26^. However, because of the resistance to HER2 inhibitor trastuzumab or the negative expression of HER2 in tumor tissues, the usage of this target agent is still limited^27–29^. So, identifying novel therapeutic targets in gastric cancer is essential.

The Hedgehog pathway is important in the oncogenesis of many human tumors^7–10^. Promoting Gli zinc-finger transcription factors translocate into nucleus, especially Gli1 is associated with tumor proliferation, metastasis and drug resistance^14–16^. In our study, we detected 67 gastric cancer tissues and showed Gli1 was related to several clinicopathological factors, such as depth of tumor invasion, lymph node metastasis and TNM staging.

Our study showed the expression levels of Gli1 and HER2 were significantly higher in gastric cancer than in para-cancer tissues. As reported, there is a significant association between the expression of Gli1 and HER2^21^. Activation of Hedgehog pathway is a multiple process and the level of Gli1 affected by several signal pathway through the crosstalk between them. A potential SMO-independent crosstalk is identified between PI3K-Akt-mTOR and Gli1^21,30–33^. Meanwhile, PI3K-Akt is the main downstream of HER2. Thus, HER2 may regulate the expression level of Gli1 by this uncanonical pathway. To confirm this hypothesis, at first, we investigated the effect of HER2 inhibitor. After adding trastuzumab to gastric cancer cells, the expression level of Gli1 decreased while SMO remained unchanged. This suggests HER2 may regulate Gli1 via SMO-independent way. Then we tested the expression of p-Akt, p-mTOR, p-p70S6k with the same treatment, and found they all decreased significantly. This means HER2 regulates its main downstream pathway PI3K-Akt-mTOR^34,35^.Then we added rapamycin to gastric cancer cells, the expression level of p-p70S6k and Gli1 decreased while SMO remained unchanged. This means mTOR interacts with Gli1 via the crosstalk. In conclusion, HER2-Akt-mTOR-S6K-Gli1 axis may be one of the pathways which HER2 regulates Gli1.

Since the interaction between Gli1 and HER2, we assessed the influence of Hedgehog pathway and HER2 inhibitors exerted on the viability of cells. Our research found the viability of cells was reduced after treated with trastuzumab or vismodegib distinctively. Moreover, the combination of trastuzumab and vismodegib has a synthesis impact on depressing cell viability.

Some previous studies considered that the high expression level of Gli1 and HER2 indicates a poor prognosis^17,18,36,37^. Whereas, in survival analysis of the 67 patients after surgery according to gastric cancer tissues IHC outcome, the differential expression of Gli1 related to different survival time (*P* < 0.05) while the HER2 didn’t (*P* = 0.170). Besides, higher Gli1 expression also leads to poorer survival time in HER2-positve patients, which is in line with the study Liu done in breast cancer^20^. Interestingly, the survival time of patients also decreased when they had a high expression of Gli1, if their basal HER2 expression is negative. It means, Gli1 expression is related to the survival time of patients. No matter the expression level of HER2, when the Gli1 expression higher, the prognosis of gastric cancer patients becomes poorer. To further explore the influence factors of the survival duration of gastric cancer patients after operation, we conducted univariate analysis and multivariate analysis, which showed venous invasion, HER2 expression, Gli1 expression were independent prognostic factors for the survival in gastric cancer patients, especially Gli1 expression. Subgroup analysis indicated that patients with Gli1 positive expression could have a significant poorer survival than others at the same depth of tumor invation (stage T3-T4), lymph node metastasis (positive) or same TNM stage (stage III-IV). In conclusion, Gli1 is an important prognosis factor of gastric cancer.

Furthermore, we confirmed it *in vitro* and *in vivo*. Firstly, we investigated the ability of cell proliferation and migration after knockdown of Gli1 in SGC7901 cells. And, the ability of cells were restricted distinctly. It explained the role Gli1 plays in the tumor growth and metastasis^38–40^. Then, 3-4 week nude mice were selected. And cells treated with control-shRNA or Gli1-shRNA were injected into subcutaneous tissues respectively. We considered the basal expression of HER2 in nude mice was similar. As expected, mice injected with Gli1-shRNA cells got a slower tumor growth and a steady weight relatively. Thus, we conclude that decreasing the Gli1 expression leads a benefit no matter the expression level of HER2. It suggests that the synthetic therapy of Gli1 suppressor and trastuzumab may act as an effective therapy of gastric cancer.

Taken together, the novel finding in this study, HER2 relates the expression of Gli1, and HER2 may regulates Gli1 in SMO-independent pathway via HER2-Akt-mTOR-S6K-Gli1 axis. There was negative influence of Gli1 on survival, and the impact is independent on the expression of HER2. Inhibiting the expression level of Gli1 leads to a great benefit *in vitro* and *in vivo*. It suggests that Gli1 may act as a novel gastric cancer suppressor. In HER2-positive patients, Gli1 inhibitor combined with trastuzumab may be an ideal choice. Meanwhile, if the HER2 expression is negative, the patients also can benefit from the use of Gli1 inhibitor. These evidence may highlight a new therapeutic strategies to gastric cancer by Gli1 inhibitor applied.

## Methods

### Patient information and follow up

A total of 67 surgically resected gastric carcinoma cases were obtained from Suzhou Municipal Hospital (Suzhou, China) from January 2008 to December 2010. All patients meet the criterial: (1)Tumor was confirmed to be gastric adenocarcinomar by pathological examination. (2)None of them received radiotherapy or chemotherapy before operation. (3) Everyone has complete clinical data and was available to be follow up. (4) All patients written informed consent. After surgery, each patient received a follow-up regularly. Until 60 months, the patients who remained alive record as 60 months.

### Preparation of human tissue samples

Paired human gastric cancer tissues and adjacent normal tissues were collected from surgically resected patients at the Department of General Surgery, the Suzhou Municipal Hospital (Suzhou, China) from 2008 to 2010. The study was approved by the Independent Ethics Committee of Suzhou Municipal Hospital. Meanwhile, we confirm that all experiments were performed in accordance with relevant guidelines and regulations.

### Immunohistochemistry

Fix the surgical specimens with formalin and embed them with paraffin. Then the tissues were cut into 5 μm. After dewaxing and rehydrating, sections were incubated with the polyclonal antibodies of Gli1 or HER2 (Cell Signaling Technology, USA; 1:200 dilution) at the room temperature for 2 or 3 hours. The process was performed with the tissue staining kit (Zhongshan Biotechnology, Beijing, China) following the manufacturer’s protocol. Two researchers got the outcome of immunostaining respectively. Five 200 × random regions were analyzed and classified into five levels according to the percentage of positively staining cells per section: absent, 0~5%; 1, 6~25%; 2, 26~50%; 3, 51~75%; 4, >75%. We deemed the staining intensity as follows: 0 (negative); 1 (weak); 2 (moderate); 3 (strong). The percentage and intensity scores were then multiplied to obtain a total score (staining score = percentage score × intensity score). For the staining score, 0 was deemed as (−), 1~4 as (+), 5~8 as (++), 9~12 as (+++). Eventually, we obtained a final average score, and consider (−) or (+) as negative, (++) or (+++) as positive^41^.

### Cell culture

Human gastric cancer cell lines SGC7901, obtained from the Cell Bank of the Chinese Academy of Sciences (Shanghai, China), were grown in RPMI Medium 1640 (Hyclone) supplemented with 10% fetal bovine serum (Gibco) and 1% penicillin/streptomycin (Gibco) and cultured at 37 °C calorstat with 5% CO_2_.

### Transfection of gastric cancer cell lines

SGC7901 cell line stably expressing Gli1-specific shRNA or scrambled control-shRNA were constructed using a lentiviral shRNA technique. The sequences specific for human Gli1 (5′-CUCCACAGGCAUACAGGAU-3′) selected to inhibit the target gene expression were synthesized by Gene Pharma (Shanghai, China). The lentivirus was titrated to 1 * 10^9^ TU/ml. SGC7901 cells were cultured at the concentration of 5 * 10^4^/well in a six-well dish for 24 h, then we added lentivirus to the culture medium. After transfection, puromycin was used to screening cells 72 hour later to form the stable cell line. The efficiency of Gli1-shRNA was detected by fluorescence microscope and Western blot.

### Protein extraction and western blotting

Whole protein extracted were lysed 30 min with RIPA lysis buffer (SigmaAldrich) according to manufacturer’s protocol. Protein collected were stored at −80 °C or used for the next immunoblot. Each sample was separated by SDS-PAGE and transferred to the nitrocellulose membranes (Schleicher & Schuell). Then, the bands incubated with the polyclonal antibody (Cell Signaling Technology) overnight after blocking with 5% non-fat milk for 1 hour at room temperature, and incubated conjugated second antibodies for 1 hour at room temperature. The images of bands were performed by chemiluminescence and results were quantified by ImageJ software.

### Cell viability assay

Cell viability was determined by MTT assays kit (Amresco, USA) according to the manufacturer’s recommendations. Cells seeded in 96-well plates and added MTT solution mixed with complete medium. After incubation for 4 hours, solution was exchanged of 150 μl DMSO. Set aside for ten minutes into the atmosphere of 37 °C and measure the absorbance at 490 nm. Each treatment had five replicate wells.

### Cell migration assays

The ability of cell migration was evaluated with 24-well transwell plate (Corning Incorporated, USA). In each well, cells resuspended in serum-free RPMI 1640 were plated in the upper chambers. And the lower chambers were added 500 μl RPMI 1640 mixed with 10% FBS. Cells that had invaded were stained with 0.5% crystal violet after 24 h incubation. Then, the cells were recorded as pictures via microscopy (200×). Quantify the level of migration by counting the invaded cells in five random regions per specimen.

### Xenografts

16 Nude mice (BALB/c, SPF grade, 16–18 g, 3–5 weeks old, male), were purchased from Shanghai SLRC laboratory Animal Co (Shanghai, China) and housed in a pathogen-free and 12 h light/dark cycle room. All nude mice were injected gastric cancer cells subcutaneously (5*10^6^ cells per mouse). All nude mice were divided into 2 groups (n = 8 mice per group) after feed 3 days according the weight and recorded the day as 0. Then, they were injected with control-shRNA and Gli1-shRNA cells respectively. We recorded body weight and tumor size twice a week. Meanwhile. Mice were killed at the day 22, and tumor were removed for further detecting. All animal experimental procedures were approved by the Animal Ethics Committee of the Suzhou Municipal Hospital (Suzhou, China). Meanwhile, we confirm that all experiments were performed in accordance with relevant guidelines and regulations.

### Statistical analysis

All experiments presented were repeated for three times. Results were expressed as means ± S.E.M. Student’s t-test was used to test the two groups. While the analysis of IHC were performed by chi-square statistical test. Survival durations were calculated using the Kaplan–Meier method and compared by the log-rank test. All parameters that were discovered to be significant on univariate analysis by the Cox proportional hazard model were then went into multivariate survival analysis. *P* < 0.05 was considered to suggest a statistically significant difference. The bars were draw with graphpad prism 5, and pictures were combined by Microsoft power point (PPT).

### Availability of data and materials

Data are stored by the corresponding author of this paper and are available upon request.

### Consent for Publication

All the individuals provided written informed consent prior to enrolling in the study, and the study was approved by the Ethics Committees of Suzhou Municipal Hospital.

## Electronic supplementary material

Supplementary information

## References

[1] Siegel RL, Miller KD, Jemal A (2017). Cancer Statistics, 2017. CA: a cancer journal for clinicians.

[2] Colquhoun A (2015). Global patterns of cardia and non-cardia gastric cancer incidence in 2012. Gut.

[3] Lee YC (2016). Association Between Helicobacter pylori Eradication and Gastric Cancer Incidence: A Systematic Review and Meta-analysis. Gastroenterology.

[4] Szakacs G, Paterson JK, Ludwig JA, Booth-Genthe C, Gottesman MM (2006). Targeting multidrug resistance in cancer. Nature reviews. Drug discovery.

[5] Parker RJ, Eastman A, Bostick-Bruton F, Reed E (1991). Acquired cisplatin resistance in human ovarian cancer cells is associated with enhanced repair of cisplatin-DNA lesions and reduced drug accumulation. The Journal of clinical investigation.

[6] Holohan C, Van Schaeybroeck S, Longley DB, Johnston PG (2013). Cancer drug resistance: an evolving paradigm. Nat Rev Cancer.

[7] Dahmane N, Lee J, Robins P, Heller P, Ruiz i Altaba A (1997). Activation of the transcription factor Gli1 and the Sonic hedgehog signalling pathway in skin tumours. Nature.

[8] Epstein EH (2008). Basal cell carcinomas: attack of the hedgehog. Nat Rev Cancer.

[9] Gulino A, Di Marcotullio L, Ferretti E, De Smaele E, Screpanti I (2007). Hedgehog signaling pathway in neural development and disease. Psychoneuroendocrinology.

[10] Berman DM (2003). Widespread requirement for Hedgehog ligand stimulation in growth of digestive tract tumours. Nature.

[11] Hui CC, Angers S (2011). Gli proteins in development and disease. Annu Rev Cell Dev Biol.

[12] Katoh Y, Katoh M (2009). Hedgehog target genes: mechanisms of carcinogenesis induced by aberrant hedgehog signaling activation. Curr Mol Med.

[13] Ng JM, Curran T (2011). The Hedgehog’s tale: developing strategies for targeting cancer. Nat Rev Cancer.

[14] Amable L, Fain J, Gavin E, Reed E (2014). Gli1 contributes to cellular resistance to cisplatin through altered cellular accumulation of the drug. Oncology reports.

[15] Ahmad A (2013). Inhibition of Hedgehog signaling sensitizes NSCLC cells to standard therapies through modulation of EMT-regulating miRNAs. Journal of hematology & oncology.

[16] Giroux Leprieur, E. *et al*. Sonic Hedgehog Pathway Activation Is Associated With Resistance to Platinum-Based Chemotherapy in Advanced Non-Small-Cell Lung Carcinoma. *Clinical lung cancer*, 10.1016/j.cllc.2015.12.007 (2015). 10.1016/j.cllc.2015.12.007 26762562

[17] Lopez-Guerrero JA (2006). HER2 amplification in recurrent breast cancer following breast-conserving therapy correlates with distant metastasis and poor survival. International journal of cancer. Journal international du cancer.

[18] Tovey SM (2009). Poor survival outcomes in HER2-positive breast cancer patients with low-grade, node-negative tumours. Br J Cancer.

[19] Alvarado-Cabrero, I. *et al*. Immunohistochemical assessment of HER2 expression in gastric cancer. A clinicopathologic study of 93 cases. *Cirugia y cirujanos*, 10.1016/j.circir.2016.11.016 (2017). 10.1016/j.circir.2016.11.016 28069112

[20] Liu S (2016). Nuclear Gli1 expression is associated with pathological complete response and event-free survival in HER2-positive breast cancer treated with trastuzumab-based neoadjuvant therapy. Tumour biology: the journal of the International Society for Oncodevelopmental Biology and Medicine.

[21] Kebenko M (2015). ErbB2 signaling activates the Hedgehog pathway via PI3K-Akt in human esophageal adenocarcinoma: identification of novel targets for concerted therapy concepts. Cell Signal.

[22] Wang Y (2012). The crosstalk of mTOR/S6K1 and Hedgehog pathways. Cancer cell.

[23] Ferlay J (2015). Cancer incidence and mortality worldwide: sources, methods and major patterns in GLOBOCAN 2012. International journal of cancer. Journal international du cancer.

[24] Van Cutsem E, Ducreux M (2016). Colorectal and gastric cancer in 2015: The development of new agents and molecular classifications. Nature reviews. Clinical oncology.

[25] Rajadurai, P., Fatt, H. K. & Ching, F. Y. Prevalence of HER2 Positivity and Its Clinicopathological Correlation in Locally Advanced/Metastatic Gastric Cancer Patients in Malaysia. *Journal of gastrointestinal cancer*, 10.1007/s12029-017-9921-1 (2017). 10.1007/s12029-017-9921-1 PMC5948243 28124769

[26] Kelly CM, Janjigian YY (2016). The genomics and therapeutics of HER2-positive gastric cancer-from trastuzumab and beyond. Journal of gastrointestinal oncology.

[27] Qiu MZ (2014). HER2-positive patients receiving trastuzumab treatment have a comparable prognosis with HER2-negative advanced gastric cancer patients: a prospective cohort observation. International journal of cancer. Journal international du cancer.

[28] Horii N, Morioka D, Yamaguchi K, Sato Y (2016). [Remarkable Response to Trastuzumab Observed in a Case of Gastric Cancer with HER2-Negative Conversion]. Gan to kagaku ryoho. Cancer & chemotherapy.

[29] Bilici A (2014). Modified docetaxel and cisplatin in combination with capecitabine (DCX) as a first-line treatment in HER2-negative advanced gastric cancer. Asian Pacific journal of cancer prevention: APJCP.

[30] Ke, Z., Caiping, S., Qing, Z. & Xiaojing, W. Sonic hedgehog–Gli1 signals promote epithelial–mesenchymal transition in ovarian cancer by mediating PI3K/AKT pathway. *Medical Oncology* **32**, 10.1007/s12032-014-0368-y (2014). 10.1007/s12032-014-0368-y 25432698

[31] Kern D (2015). Hedgehog/GLI and PI3K signaling in the initiation and maintenance of chronic lymphocytic leukemia. Oncogene.

[32] Stecca B (2007). Melanomas require HEDGEHOG-GLI signaling regulated by interactions between GLI1 and the RAS-MEK/AKTpathways. Proceedings of the National Academy of Sciences.

[33] Zuo M (2015). Novel therapeutic strategy targeting the Hedgehog signalling and mTOR pathways in biliary tract cancer. British Journal of Cancer.

[34] Choi Y (2016). HER2-induced metastasis is mediated by AKT/JNK/EMT signaling pathway in gastric cancer. World journal of gastroenterology.

[35] Sukawa Y (2014). HER2 expression and PI3K-Akt pathway alterations in gastric cancer. Digestion.

[36] Xu L (2010). Gli1 promotes cell survival and is predictive of a poor outcome in ERalpha-negative breast cancer. Breast cancer research and treatment.

[37] Merkin, R. D. *et al*. Keratin 17 is Overexpressed and Predicts Poor Survival in ER-/HER2- Breast Cancer. *Human pathology*, 10.1016/j.humpath.2016.10.006 (2016). 10.1016/j.humpath.2016.10.006 27816721

[38] Wang H (2012). Study on the skip metastasis of axillary lymph nodes in breast cancer and their relation with Gli1 expression. Tumour biology: the journal of the International Society for Oncodevelopmental Biology and Medicine.

[39] Ahmed AR (2016). HER2 expression is a strong independent predictor of nodal metastasis in breast cancer. Journal of the Egyptian National Cancer Institute.

[40] Adamczyk A (2017). Proteins Involved in HER2 Signalling Pathway, Their Relations and Influence on Metastasis-Free Survival in HER2-Positive Breast Cancer Patients Treated with Trastuzumab in Adjuvant Setting. Journal of Cancer.

[41] Yang K (2015). Short hairpin RNA- mediated gene knockdown of FOXM1 inhibits the proliferation and metastasis of human colon cancer cells through reversal of epithelial-to-mesenchymal transformation. J Exp Clin Cancer Res.

