# Supplementary material for: Correlation of Gli1 and HER2 expression in gastric cancer: Identification of novel target
Source: Sci Rep. 2018 Jan 10;8:397. doi: 10.1038/s41598-017-17435-6 (PMC5762756; doi:10.1038/s41598-017-17435-6)
Supplement: Supplementary file 1 — Supplementary information [file 41598_2017_17435_MOESM1_ESM.pdf]

Correlation of Gli1 and HER2 expression in gastric cancer: Identification of novel target

Xinyu Shao[1], Xiaoyi Kuai[1], Zhi Pang[1], Liping Zhang[1], Longyun Wu[1], Lijuan Xu[1], Chunli Zhou[1,*]

1.The Affiliated Suzhou Hospital of Nanjing Medical University, Suzhou 215006.

*corresponding author,email:zhouchunli079@163.com

Supplementary information

Supplementary figures

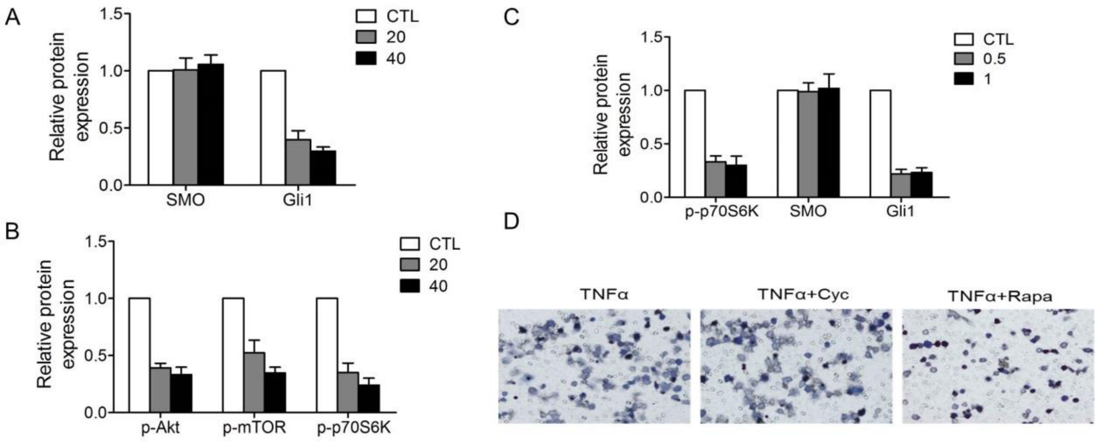

Figure S1. HER2 regulates Gli1 via Akt-mTOR pathway. (A) Grey levels of Figure 5A: the expression of SMO and Gli1 after SGC-7901 treated with Trastuzumab for 48h. Grey levels were evaluated as mean ± S.E.M. (n = 3). (B) Grey levels of Figure 5B: the expression of p-Akt, p-mTOR, p-70S6K after SGC-7901 treated with Trastuzumab for 48h. Grey levels were evaluated as mean ± S.E.M. (n = 3). (C) Grey levels of Figure 5C: the expression of p-70S6K, SMO and Gli1 after SGC-7901 treated with rapamycin for 48h. Grey levels were evaluated as mean ± S.E.M. (n = 3). (D) Transwell assay result of Figure 5F:SGC7901 cells treated with TNFα (5ng/ml), TNFα (5ng/ml)and cyclopamine (1μM) or TNFα (5 ng/ml) and rapamycin (0.5μM) for 24h.
